# Biodegradable Polymers Influence the Effect of Atorvastatin on Human Coronary Artery Cells

**DOI:** 10.3390/ijms17020148

**Published:** 2016-01-22

**Authors:** Anne Strohbach, Robert Begunk, Svea Petersen, Stephan B. Felix, Katrin Sternberg, Raila Busch

**Affiliations:** 1 Department of Internal Medicine B (Cardiology), University Medicine Greifswald, Ferdinand-Sauerbruch-Strasse, 17475 Greifswald, Germany; felix@uni-greifswald.de (S.B.F.); buschr@uni-greifswald.de (R.B.); 2 DZHK (German Centre for Cardiovascular Research), Partner Site Greifswald, University Medicine Greifswald, 17475 Greifswald, Germany; 3 Institute of Pharmacology, University Medicine Greifswald, Felix-Hausdorff-Strasse 3, 17477 Greifswald, Germany; begunk@gmx.de; 4 Faculty of Engineering and Informatics, Osnabrück University of Applied Sciences, Albrechtstrasse 30, 49076 Osnabrück, Germany; s.petersen@hs-osnabrueck.de; 5 Research & Development, Aesculap AG, Am Aesculap Platz, 78532 Tuttlingen, Germany; katrin.sternberg@aesculap.de

**Keywords:** atorvastatin, poly(l-lactide), biodegradable polymer, human coronary artery endothelial cells, human coronary artery smooth muscle cells

## Abstract

Drug-eluting stents (DES) have reduced in-stent-restenosis drastically. Yet, the stent surface material directly interacts with cascades of biological processes leading to an activation of cellular defense mechanisms. To prevent adverse clinical implications, to date almost every patient with a coronary artery disease is treated with statins. Besides their clinical benefit, statins exert a number of pleiotropic effects on endothelial cells (ECs). Since maintenance of EC function and reduction of uncontrolled smooth muscle cell (SMC) proliferation represents a challenge for new generation DES, we investigated the effect of atorvastatin (ATOR) on human coronary artery cells grown on biodegradable polymers. Our results show a cell type-dependent effect of ATOR on ECs and SMCs. We observed polymer-dependent changes in IC_50_ values and an altered ATOR-uptake leading to an attenuation of statin-mediated effects on SMC growth. We conclude that the selected biodegradable polymers negatively influence the anti-proliferative effect of ATOR on SMCs. Hence, the process of developing new polymers for DES coating should involve the characterization of material-related changes in mechanisms of drug actions.

## 1. Introduction

In the past decades, rapid progress in stent development eventually led to the launching of drug-eluting stents (DES), which facilitate local drug delivery to the injured vessel wall. Since then, local drug delivery has emerged as a very promising approach to manage in-stent-restenosis [1]. However, despite their excellent efficacy regarding the inhibition of in-stent-restenosis, first generation DES were soon associated with late stent thrombosis and delayed wound healing [2,3,4,5], an effect due to the anti-proliferative actions of released drugs targeting not only smooth muscle cells (SMCs) but also, unintentionally, endothelial cells (ECs) as well [6].

The pathology of stent thrombosis has not been fully elucidated, yet. However, studies of patients dying from late stent thrombosis—after implantation of first generation sirolimus- or paclitaxel-eluting stents—have shown that these DES are associated with delayed wound healing and impaired re-endothelialisation [7]. This might be due, at least in part, to the polymer coating, which facilitates drug release from the stent struts but remains in the vessel long after drug elution is complete. However, the stent surface functions as an interface between the medical implant and either the streaming blood at the luminal site or the arterial wall at the abluminal site. Hence, the surface material directly interferes with numerous biological processes frequently leading to an activation of humoral and cellular immune responses [8,9]. The evidence is growing that permanent polymers can cause delayed wound healing, impaired endothelialisation, and hypersensitivity reactions, which all together can culminate in stent thrombosis [10,11,12]. New technologies include the use of polymer-free DES and DES with biodegradable coating matrices or fully degradable vascular scaffolds [7,13]. In this context, intensive research has been conducted to develop novel polymers with the aim to improve the biocompatibility of vascular implants. Biodegradable polylactide (PLA) and copolymers, such as poly(lactide-*co*-glycolide) (PLGA), are now being considered as stent coating platforms [14,15,16]. Recently, we have shown that a polymeric blend consisting of poly(l-lactide) (PLLA) and poly(4-hydroxybutyrate) (P(4HB)) in a ratio of 78%/22% (*w*/*w*) shows improved biocompatibility regarding re-endothelialisation, cell viability, and endothelial cell function, as well as a lower thrombogenicity, compared to commonly used coating polymers [17,18,19,20].

3-Hydroxy-3-methylglutaryl coenzyme A (HMG-CoA) reductase inhibitors (statins) are widely used to treat dyslipidemia, especially in patients with cardiovascular diseases [21,22]. Statins are well-known for their beneficial effects in both primary and secondary prevention of coronary heart disease [23]. These drugs trigger various protective effects on the cardiovascular system. These so-called “pleiotropic” effects involve the improvement of endothelial function, stabilization of atherosclerotic plaques, the decrease of oxidative stress and inflammation, and inhibition of thrombogenic response [24,25,26,27,28,29,30]. Clinical data suggest a positive impact of atorvastatin (ATOR) on the clinical outcome of patients undergoing percutaneous coronary intervention (PCI) [31].

Statins are taken up from the portal blood by transporters of organic anion transporting polypeptides (OATPs) [32]. Amongst the members of OATP family, only OATP2B1 seems to be expressed ubiquitously and turned out to be a high-affinity transport protein for ATOR with a considerably lower affinity to other statins [33]. The inhibition of HMG-CoA reductase by statins has been proposed as a potential mechanism of pleiotropic actions in the prevention of vascular damage [34]. ATOR is reported to modulate the cellularity of the artery wall by inhibiting proliferation of vascular SMCs and enhancing apoptotic cell death, which might be beneficial to retard hyperplasia and in-stent-restenosis [34,35].

We already have demonstrated a polymer-dependent endothelialisation, SMC growth, and thrombogenicity with the result that some of the commonly used coating polymers did not support several criteria for biocompatibility [19,20]. However, the investigated biodegradable polymeric blend PLLA/P(4HB) represented a promising material for biodegradable vascular scaffolds and stent coatings, although it did not show particularly limited SMC growth. It is a fact that most of the patients undergoing PCI with stent implantation are given statins (e.g., ATOR) to reduce the incidence of adverse coronary events [36]. In addition, new approaches in stent coating include the release of statins to improve re-endothelialisation and prevent uncontrolled SMC proliferation [37,38]. However, even though the first-in-man study of a simvastatin-eluting stent (SIMVASTENT) has established its safety, it has failed to provide evidence of lower-than-expected neo-intimal inhibition [39]. In this context, this *in vitro* study was designed to investigate the impact of the biodegradable coating polymers PLLA, P(4HB), and PLLA/P(4HB) on the effect of ATOR on SMC proliferation. All experiments were referred to the control surface Thermanox™ (Thermo Fisher Scientific, Darmstadt, Germany) which is known for its high biocompatibility.

## 2. Results and Discussion

Comprehensive requirements on stent coatings complicate their improvement since the polymeric material ideally should ensure an effective re-endothelialisation. Simultaneously, the polymer should effectively prevent an overgrowth of SMCs and thereby inhibit restenosis [40]. However, little is known about the factors that influence cell growth and viability on polymer surfaces, and it is not evident that one single stent polymer itself will combine all desirable aspects.

### 2.1. The Impact of Biodegradable Polymers on Vascular Cell Viability and Proliferation

**Figure 1:**
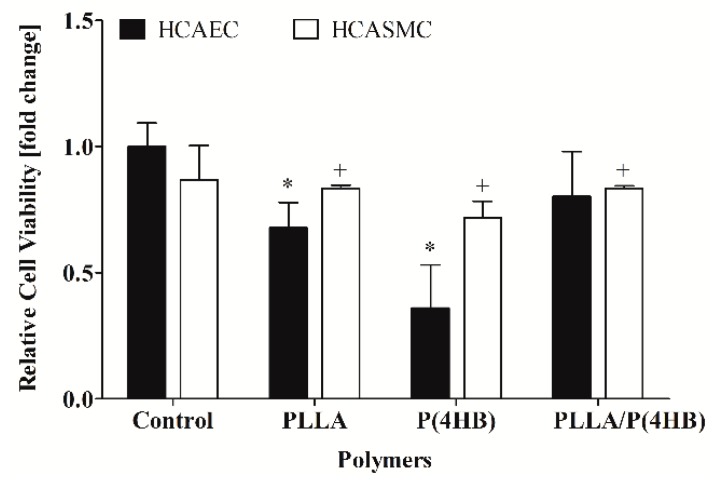
The viability of vascular cells is influenced by polymers. Human coronary artery endothelial cells (HCAEC) and human coronary artery smooth muscle cells (HCASMC) were cultured on control and polymer surfaces for six days. Cell viability was determined after incubation with AlamarBlue^®^ (Biosource, Camarillo, CA, USA). Bars show mean ± SD of six separate experiments normalized to the control with * *p* < 0.05 compared to the HCAEC control and ^+^ *p* < 0.05 compared to the HCASMC control.

In order to assess the impact of the polymers on human coronary artery endothelial cells (HCAEC) and human coronary artery smooth muscle cells (HCASMC) proliferation, AlamarBlue^®^ (Biosource, Camarillo, CA, USA) and BrdU-ELISA (Roche, Basel, Switzerland) assays were performed. Statistical analysis of the results revealed a significant reduction of endothelial viability which was caused by the polymers but not related to the cell type (*p* = 0.0001; Figure 1). HCAEC show a significantly decreased viability when grown on PLLA and P(4HB) (0.68 ± 0.1 and 0.36 ± 0.2 with *p* = 0.029, respectively). However, the polymeric blend PLLA/P(4HB) does not influence HCAEC viability significantly (0.80 ± 0.2, *p* = 0.114). Furthermore, the viability of HCASMC is also strongly affected by incubation with the different polymers. HCASMC show a significantly decreased viability when grown on either one of the three polymers (*p* = 0.029). We also found a material-dependent cell proliferation with best results on the polymeric blend PLLA/P(4HB) for both, HCAEC and HCASMC [20].

Our data show, that viability of ECs and SMCs is reduced by cultivation on polymeric surfaces. However, EC and SMC proliferation on polymers is still sufficient, when compared to a cell culture control, with no detectable preference for one cell type. Hence, there is a need to administer drugs, which are able to inhibit the growth of SMCs but also encourage EC growth.

### 2.2. The Impact of Atorvastatin (ATOR) on Vascular Cell Viability and Proliferation

When given orally, ATOR has been shown to exert a number of pleiotropic effects including improvement of EC function [29,30]. Therefore, new stent-designs include the abluminal focused release of ATOR from DES to enhance re-endothelialisation after stent implantation [37].

**Figure 2:**
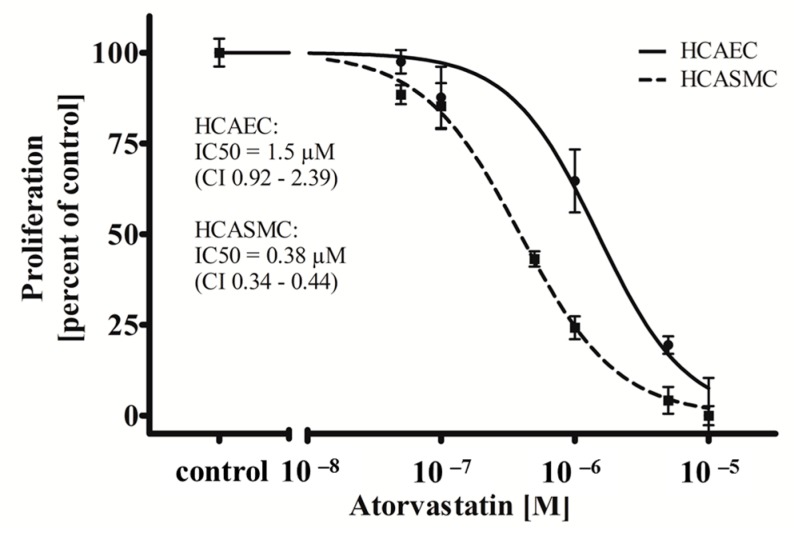
Atorvastatin (ATOR) acts cell type-specifically on human coronary artery vascular cells. HCAEC and HCASMC were treated for 48 h with the indicated range of concentrations of ATOR. Proliferation was determined after BrdU incorporation. Dots show mean ± SD of at least three separate experiments normalized to the respective control. IC_50_ values and the respective 95% confidence interval (CI) are indicated.

In this study, we show that ATOR, when administered systemically, is able to act cell type-specifically on HCAEC and HCASMC (Figure 2). While treatment with ATOR does not influence the viability of both HCAEC and HCASMC grown on a control surface (Supplemental Data 1), we observed a significant cell type-dependent (*p* = 0.0001) and dose-dependent anti-proliferative effect (*p* = 0.0001). As shown in Figure 2, ATOR inhibits HCASMC proliferation with an IC_50_ value of 0.38 µM (Confidence Interval (CI) 0.34–0.44 µM) which is in accordance with the literature [41]. Interestingly, the observed IC_50_ value of ATOR was more than threefold higher for HCAEC compared to HCASMC (1.5 µM; (CI 0.92–2.39 µM); *p* = 0.0001), which has also been described [37].

Based on these results, one may assume that the cell type-specific anti-proliferative impact of ATOR on SMCs, given either systemically or administered locally, might exert beneficial effects on both re-endothelialisation and SMC proliferation.

### 2.3. The Impact of ATOR on Vascular Cell Viability and Proliferation

Experience has shown that the process of re-endothelialisation on polymeric surfaces is not only determined by the administration of diverse drugs but also by the polymer itself. Thus, we decided to determine the influence of ATOR on HCAEC and HCASMC grown on polymer surfaces. Regarding the control surface, incubation of HCAEC and HCASMC with 0.1 µM ATOR results in a 40% reduction of HCASMC growth without having an impact on HCAEC proliferation (*p* = 0.001). To test its effects on HCAEC and HCASMC grown on the three different biodegradable stent coating polymers, we chose a concentration of 0.1 µM ATOR—a concentration, which is well within the range of plasma concentrations of subjects receiving ATOR orally [42].

**Figure 3:**
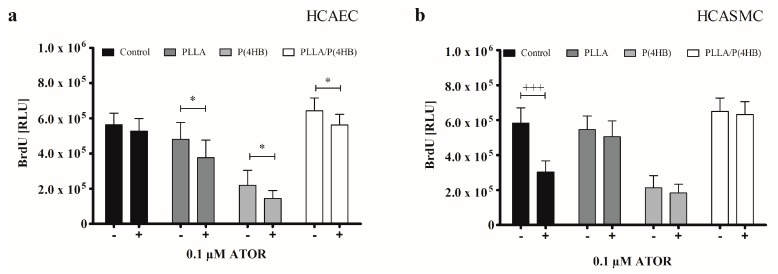
Polymers attenuate anti-proliferative actions of ATOR on SMCs. (**a**) HCAEC were cultured on polymer surfaces in the absence of ATOR (−) or presence of 0.1 µM ATOR for 48 h (+); (**b**) HCASMC were cultured on polymer surfaces and treated for 48 h with 0.1 µM ATOR (+). Proliferation was determined after BrdU incorporation. Bars show mean ± SD of three independent experiments with * *p* < 0.05 compared to the corresponding HCAEC control and ^+++^ *p* < 0.001 compared to the HCASMC control.

Our results show, that polymers influenced incubation of HCAEC with 0.1 µM ATOR (Figure 3a; *p* = 0.0001). While ATOR treatment had no effect on the proliferation of HCAEC grown on the control surface (564.434 ± 65.540 *vs.* 527.485 ± 70.763; *p* = 0.267), growth was significantly inhibited on each of the three polymer surfaces (PLLA: 480.967 ± 95.495 *vs.* 376.431 ± 100.263; *p* = 0.038, P(4HB): 219.107 ± 85.852 *vs.* 145.018 ± 45.400; *p* = 0.036, PLLA/P(4HB): 643.432 ± 72.110 *vs.* 561.513 ± 61.718; *p* = 0.020). As for the control surface, HCASMC proliferation was significantly decreased by 50% compared to HCASMC treated with 0.1 µM ATOR (Figure 3b: 581.855 ± 88.535 *vs.* 303.119 ± 64.264; *p* < 0.0001). Yet, this effect was not observed on PLLA, P(4HB), and PLLA/P(4HB) surfaces.

**Figure 4:**
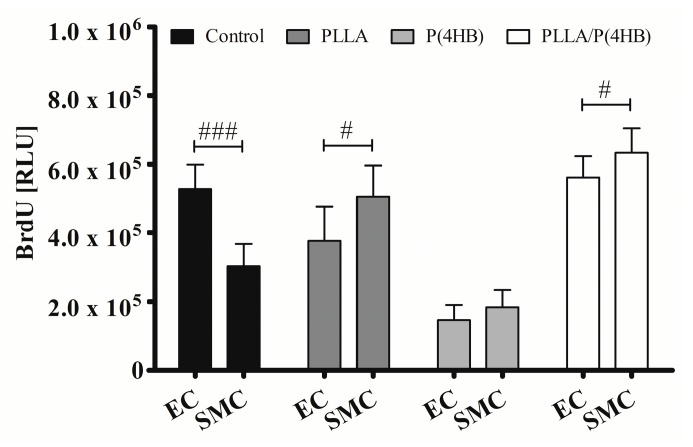
Polymers attenuate cell type-specific anti-proliferative effects of ATOR on vascular cells. HCAEC (EC) and HCASMC (SMC) were cultured on polymer surfaces and treated for 48 h with 0.1 µM ATOR. Proliferation was determined after BrdU incorporation. Bars show mean ± SD of at least five separate experiments with ^#^ *p* < 0.05 and ^###^ *p*< 0.001.

Our results reveal a cell type-specific and anti-proliferative effect of ATOR on vascular coronary artery cells. This effect seems to be abolished when HCASMC were grown on polymeric surfaces. Figure 4 shows that the polymer influences the action of ATOR on HCASMC and HCAEC (*p* = 0.0001). The control surface shows an almost 50% decrease in proliferation of ATOR-treated HCASMC compared to HCAEC (303.119 ± 64.264 *vs.* 527.485 ± 70.763; *p* < 0.0001). On PLLA and PLLA/P(4HB), 0.1 µM ATOR does not diminish SMC proliferation compared to HCAEC. In fact, SMC proliferation seems to be enhanced (PLLA: 505.327 ± 90.814 *vs.* 376.431 ± 100.263; *p* = 0.011, PLLA/P(4HB): 633.054 ± 72.308 *vs.* 561.513 ± 61.718; *p* = 0.038). On P(4HB), no cell type-specific ATOR-related effect was observed (183.560 ± 50.411 *vs.* 145.018 ± 45.400; *p* = 0.108).

Such cell type-specific effects of ATOR have been described in the literature [43]. Furthermore, it has been shown that at concentrations up to 10 µM, cell type-specific actions of ATOR are abolished and proliferation of ECs is diminished [44]. Other studies underline these findings by demonstrating that ATOR at concentrations up to 0.1 µM promotes migration of human umbilical vein ECs and endothelial progenitor cells. Higher concentrations, however, inhibited angiogenesis and migration [45].

In part, our results can be explained by the observed changes in IC_50_ values for ATOR regarding HCAEC and HCASMC grown on PLLA, P(4HB), and PLLA/P(4HB). Table 1 gives an overview of IC_50_ values (CI). We found a significant, polymer-dependent alteration in IC_50_ values for HCASMC (*p* = 0.024) with the highest IC_50_ for PLLA/P(4HB) (0.86 µM; (CI 0.92–2.39 µM)) indicating a lower inhibition potency. This means that a two-fold higher concentration of ATOR is required to inhibit HCASMC proliferation by 50%. Likewise, HCAEC show a tendency to reduced ATOR IC_50_ values especially on PLLA and P(4HB). Even though this reduction was not found to be statistically significant (*p* = 0.138) this might be a factor contributing to the observed reduction of HCAEC growth on polymers when incubated with ATOR.

**Table 1:** IC_50_ of ATOR on HCASMC and HCAEC grown on different polymer surfaces. IC_50_ values were calculated from sigmoidal dose-response curves as the concentration of ATOR causing 50% reduction in cell growth. The 95% confidence intervals (CI) are indicated.

Polymer	HCASMC IC_50_ (µM)	CI	HCAEC IC_50_ (µM)	CI
**Thermanox™**	0.38	0.34–0.44	1.49	0.93–2.39
**PLLA**	0.49	0.38–0.65	0.79	0.12–5.30
**P(4HB)**	0.53	0.35–0.80	0.67	0.15–2.99
**PLLA/P(4HB)**	0.86	0.50–1.50	1.20	0.51–2.83

### 2.4. Biodegradable Polymers Affect ATOR-Uptake

Our results show a lack of anti-proliferative effects of ATOR on HCASMC grown on polymeric poly(l-lactide)-based surfaces. In fact, it seems that proliferation of SMCs is enhanced on polymer surfaces PLLA and PLLA/P(4HB). In order to explain the effects of ATOR on coronary arterial cells, we investigated the uptake of radio-labelled ^3^H-ATOR in HCAEC and HCASMC.

**Figure 5:**
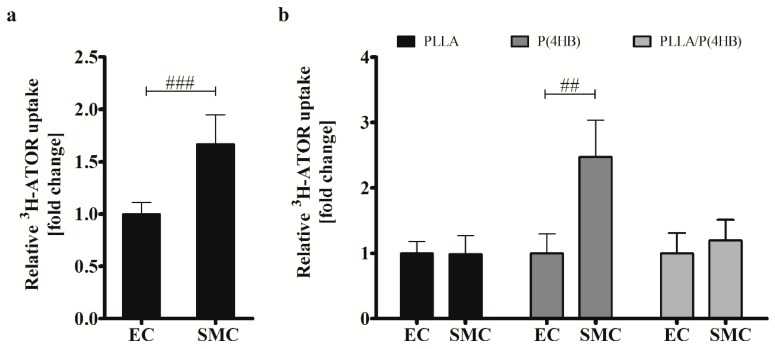
Uptake of ^3^H-ATOR is cell type-specific and polymer-dependent. (**a**) HCAEC (EC) and HCASMC (SMC) were treated for 20 min with radio-labelled ^3^H-ATOR. Bars show mean ± SD of at least 10 separate experiments normalized to HCAEC grown on Thermanox™ (Thermo Fisher Scientific, Darmstadt, Germany) (^###^ *p* < 0.001); (**b**) HCAEC (EC) and HCASMC (SMC) were grown on polymer surfaces prior to ^3^H-ATOR treatment. Data are shown as the percentage of the ATOR-uptake in ECs. Each bar shows mean ± SD of at least three separate experiments. In each case, SMC data were normalized to their respective ECs (^##^ *p* < 0.01).

Regarding the control surface, ATOR uptake is 1.66-fold higher in HCASMC than in HCAEC (*p* = 0.001; Figure 5a) causing the differential effect of ATOR on the proliferation of arterial ECs and SMCs (compare Figure 2). On biodegradable polymers, this effect seems to be abolished which is in accordance with our above-presented results regarding cell growth and IC_50_ values. In fact, we found a globally increased uptake of radio-labelled ATOR in HCAEC grown on polymer surfaces (Supplemental Data 2). While ATOR-uptake is significantly increased in HCAEC on all polymers tested (*p* = 0.002), we found the strongest increase in HCASMC grown on P(4HB). However, ATOR at a concentration of 0.1 µM does not affect HCASMC proliferation on P(4HB) (compare Figure 4) even though ATOR-uptake is increased by 2.5-fold (*p* = 0.01). Additionally, the 1.4-fold enhanced IC_50_ value of HCASMC grown on P(4HB) indicates a lower inhibition potency of ATOR (compare Table 1).

### 2.5. Biodegradable Polymers Influence Gene Expression of HCASMC

**Figure 6:**
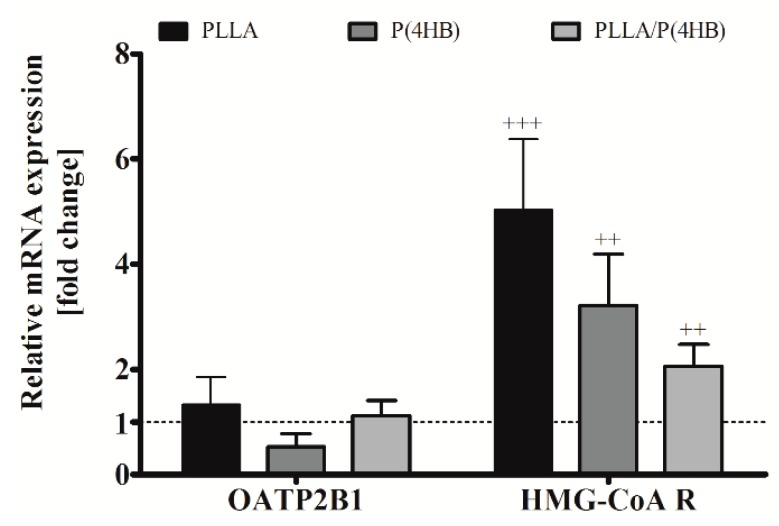
Polymer-dependent gene expression of HCASMC. HCASMC were cultured on control and polymer surfaces for six days. Subsequently, cells were harvested and prepared for quantitative RT-PCR. Bars show mean ± SD of at least six separate experiments normalized to the Thermanox™ (Thermo Fisher Scientific, Darmstadt, Germany) control (dotted line) (^++^ *p* < 0.01 and ^+++^ *p* < 0.001).

Once transported into the cell by cationic transporters of the OATP family, statins act as direct inhibitors of the enzyme HMG-CoA reductase. Therefore, we investigated the influence of polymers on gene expression of the cationic transporter OATP2B1, which is expressed in cardiac tissue [33], and HMG-CoA reductase in HCASMC (Figure 6). We could not find a significant influence of the polymer on OATP2B1 gene expression in HCASMC. However, aiming at the mismatch, enhanced ATOR-uptake and reduced inhibition potency, we also investigated the gene expression of HMG-CoA reductase. The expression of HMG-CoA reductase was significantly increased by the cultivation of HCASMC on polymer surfaces for all polymers tested (Figure 6, *p* < 0.001) with the highest expression on PLLA and P(4HB) (5.02 ± 1.36; *p* = 0.001 and 3.22 ± 0.97; *p* = 0.003, respectively).

It has been described recently that changes in the micromechanical environment of cells may regulate cellular functions relevant to development, homeostasis, and disease [46,47,48,49]. Drug responses of various cells grown on soft, stiff, and rigid substrates have shown a broad range of possible material-related effect mechanisms (reviewed in [50,51]). Additionally, it has been shown that rigid substrates limit anti-proliferative effects of mytomycin C on mesenchymal stem cells due to stronger adhesion to stiffer substrates [52,53]. PLLA and PLLA based polymers are extremely stiff materials [53] and are therefore likely to be perceived by cells as rigid [51,54]; they may thus influence drug-response of HCAEC and HCASMC to ATOR as well.

### 2.6. Systemic Drug Administration versus Local Drug Delivery

**Figure 7:**
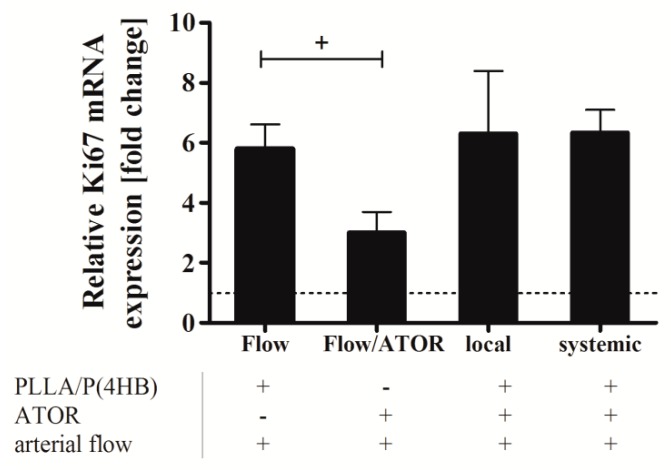
Ki67 expression in human coronary artery smooth muscle cells (HCASMC) after local and systemic atorvastatin (ATOR) administration. HCASMC were cultured on polycarbonate membranes and placed on ATOR-releasing PLLA/P(4HB) surfaces or PLLA/P(4HB) films. Cells were perfused at 20 dyne/cm^2^ for 3 h using a parallel plate flow chamber. Perfusion was performed in basal medium supplemented with 2% FBS. Plus (+) and minus (−) indicate the presence or absence of a component indicated below the bar chart. After flow exposure, cells were detached and prepared for quantitative RT-PCR. Bars show mean ± SD of at least three separate experiments normalized to HCASMC kept under static conditions in basal medium supplemented with 2% FBS (^+^ *p* < 0.05).

To determine the effect of ATOR on HCASMC growth, when locally released from a PLLA/P(4HB) platform, we assessed expression of Ki67. Ki67 is a nuclear protein, which is expressed during all stages of the cell cycle except the resting phase. It has been described as a well-established marker of proliferation in various tumor types [55]. To validate the correlation of Ki67 expression and proliferation in SMCs we determined mRNA-levels of Ki67 in serum-starved HCASMC (basal medium supplemented with 0.5% FBS), control HCASMC (basal medium supplemented with 2% FBS), and growing HCASMC (growth medium supplemented with 10% FBS) (Supplemental Data 3) and compared these results to a commercially available BrdU-ELISA (Roche, Basel, Switzerland). Since the results fitted well, Ki67 was considered a sufficient marker to assess proliferation of HCASMC under flow conditions. Figure 7 shows the relative Ki67 mRNA level in HCASMC grown on PLLA/P(4HB) surfaces without ATOR (Flow), HCASMC grown on glass cover slips and perfused with 0.1 µM ATOR (ATOR), and in HCASMC grown on PLLA/P(4HB) with systemic administration of 0.1 µM ATOR or locally released ATOR.

Under flow, the proliferation of HCASMC is elevated by the factor 5.8 compared to the control kept in 2% FBS (dotted line). Data show, that this flow-induced proliferative effect is significantly reduced by 50% (*p* = 0.021). However, the anti-proliferative effect of ATOR, either given systemically or locally, was abolished, when HCASMC were cultured on the polymeric blend PLLA/P(4HB).

The key advantage of drug-eluting stents is an effective local drug delivery of high drug doses by avoiding systemic toxicity. Over a defined period of time, the drug can be delivered with controlled kinetics, which have proven to be appropriate to the vascular healing process [56] allowing for higher local concentrations at the injury site. A recent work showed the effectiveness of the local delivery of high doses of rosuvastatin in improving re-endothelialisation and inhibiting neo-intimal formation in an *in vivo* rabbit model of vascular injury and stenting [38]. Although the authors of this study developed a promising new hybrid biodegradable nano fibrous rosuvastatin-loaded stent, various studies performed with human ECs imply that statins lose their supportive effects when given in high dosages [43,44,45]. In addition, the SIMVASTENT study did not support the expected effect of statin-release on neo-intimal proliferation and re-endothelialisation *in vivo*. Our data suggest that the stent coating cannot only influence drug release, but also the potency of the released drug by altering uptake properties and gene expression of the target cells. Here we show, that for the local delivery of ATOR, embedded in a highly biocompatible copolymer PLLA/P(4HB), is not superior to the systemic administration of ATOR regarding SMC proliferation.

## 3. Experimental Section

### 3.1. Polymer Film Preparation

The following polymeric materials were used: poly(l-lactide) (PLLA, Resomer^®^ L214, *M*_W_ = 720.000 g/mol, Boehringer Ingelheim Pharma, Ingelheim, Germany) and poly(4-hydroxybutyrate) (P(4HB), *M*_W_ = 170.000 g/mol, TephaFLEX^®^, Tepha Inc., Lexington, MA, USA). Whereas we employed a pouring process for P(4HB) and the polymeric blend composed of 78% (*w*) PLLA and 22% (*w*) P(4HB), we applied manual dip-coating for PLLA. For further details see [20]. Prior to *in vitro* testing, all films were sterilized by a common ethylene oxide sterilization process and afterward stamped out under sterile conditions into disks of 25 and 5 mm diameter.

### 3.2. Cell Culture

Primary cultures of human coronary artery endothelial cells (HCAEC) and human coronary artery smooth muscle cells (HCASMC) were purchased from Provitro GmbH, Berlin, Germany. Cells were obtained at passage 2 and cultured in endothelial cell growth medium (ECGM, Provitro GmbH, Berlin, Germany) or smooth muscle cell growth medium (SMCGM, Provitro GmbH) supplemented with 10% fetal bovine serum (FBS, Invitrogen, Carlsbad, CA, USA). Cells were used in passages 3–6 and cultured for 6 days on the polymers before treatment.

### 3.3. Cell Viability and Proliferation Assay

Cell viability was assessed by incubation with AlamarBlue^®^ (Biosource, Camarillo, CA, USA). Cells were seeded onto Ø 5 mm Thermanox™ cover slips or polymer films at a density of 6.000 cells per 96-well. AlamarBlue^®^ was added to each well as 10% of the sample volume, and cells were incubated for 16 h. The resulting fluorescence signal of resorufin was measured at an excitation wavelength of 530 nm and an emission wavelength at 590 nm using a microplate reader (Infinite^®^ 200 Pro, Tecan Deutschland GmbH, Crailsheim, Germany). Data are normalized to the control cells grown on Thermanox™ cover slips.

The relative proliferation rates were quantified using a commercially available BrdU Cell Proliferation ELISA (Roche, Basel, Switzerland). HCAEC and HCASMC were seeded as described above and cultured for 6 days in black 96-well micro plates with flat, clear bottom (Greiner Bio-One GmbH, Frickenhausen, Germany). According to the manufacturer’s instructions, 100 µM BrdU was added to each well and incubated for 24 h at 37 °C, 5% CO_2_, and 95% humidity. Cell proliferation was determined using an Infinite^®^ 200Pro microplate reader (Tecan Deutschland GmbH, Crailsheim, Germany).

### 3.4. Cell Growth Inhibition Assay

HCAEC and HCASMC were seeded in 96-well microtiter plates (Greiner, Bio-One GmbH, Frickenhausen, Germany) at a density of 6000 cells/well either on Ø = 5 mm Thermanox™ (Thermo isher Scientific, Darmstadt, Germany) cover slips or on polymer films. Drug dilutions were freshly made in ECGM and SMCGM, respectively, and serially diluted over a 0.01 up to 10 µM range of concentration. The impact of ATOR (Hartmann Analytics, Braunschweig, Germany) on proliferation was determined after 48 h performing a BrdU Cell Proliferation ELISA (Roche, Mannheim, Germany). IC_50_ values were determined to calculate sigmoidal dose-response-curves using the GraphPad© Prism 5 software (La Jolla, CA, USA).

### 3.5. Uptake of Radio-Labelled ^3^H-ATOR

HCAEC and HCASMC were seeded in 24-well microtiter plates (Greiner, Bio-One GmbH, Frickenhausen, Germany) at a density of 50,000 cells/well on polymer films respectively Thermanox™ cover slips and grown to confluence in their respective growth media. Directly before starting ATOR-uptake measurement, cells were washed with 1 mL of pre-warmed PBS. For transport experiments, the incubation buffer (NaCl 142 mM, KCl 5 mM, KH_2_PO_4_ 1 mM, CaCl_2_ 1.5 mM, MgSO_4_ 1.5 mM, Glucose 5 mM, HEPES 12.5 mM) containing 1.25 nM ^3^H-ATOR and 1 µM of unlabeled ATOR (Hartmann Analytics, Braunschweig, Germany) was pre-warmed at 37 °C. After 20 min of incubation at 37 °C uptake was interrupted by washing three times with ice-cold PBS. Cells were then lysed using 500 µL of lysis buffer (0.2 mM SDS, 5 mM EDTA). Subsequently, to complete lysis, 150 µL of the lysate were transferred into scintillation vials containing 2 mL Rotiszint^®^ eco plus (Carl Roth, Karlsruhe, Germany). Finally incorporated radioactivity was determined using a Tri-Carb Liquid Scintillation Analyzer (PerkinElmer, Rodgau, Germany).

### 3.6. Preparation of ATOR-Releasing PLLA/P(4HB) Surfaces

PLLA and P(4HB) were dissolved in chloroform yielding a concentration of 1.95 and 0.55 mg/mL, respectively. ATOR was subsequently mixed into the polymer solution to obtain a final drug amount of 17.5% (*w*) in PLLA/P(4HB). The solution was then applied to Ø 25 mm glass coverslips via a 2-component jet atomization spray coating process. The coating mass of 3.90 µg was adjusted by intermediate weighing using a microbalance (UMX5, Mettler Toledo, Giessen, Germany). Hence, glass cover slips received a total ATOR load of 1.4 µg/mm^2^ in accordance to the drug load on conventional DES, for instance the Orsiro (Biotronik, Berlin, Germany). Coated cover slips were then tempered for 13.5 h at 80 °C in a vacuum and finally sterilized by a commonly used ethylene oxide sterilization process. The ATOR load was confirmed via HPLC. Therefore, polymer films were extracted in 10, and subsequently in 4 mL methanol at 80–2 °C for 30 and 60 min respectively. 20 µL of each aliquot were injected into a Eurospher 100 C18 column, 120 × 4 mm ID (Wissenschaftlicher Gerätebau Dr.-Ing. Herbert Knauer GmbH, Berlin, Germany): column temperature 50 °C; isocratic eluent acetonitrile/water 65/35 (*v*/*v*); flow rate 1.0 mL/min and UV detection at 248 nm with calibrated measurement range 0.05–10.0 mg/L. The amount of drug released after 3 h perfusion at 20 dyn/cm^2^ in SMCBM supplemented with 2% FBS was determined by the same HPLC method and calculated as 0.17 µM.

### 3.7. Local Drug Delivery Model

Before HCASMC were seeded on Nuclepore™ track-etched polycarbonate membranes (Ø 25 mm, pore size 5 µm; Whatman GmbH, Dassel, Germany), membranes were incubated for one hour with 1 mL of 100 µM collagen I (Gibco^®^, Carlsbad, CA, USA) in 10 mL PBS^−/−^ (Gibco^®^, Carlsbad, CA, USA). After incubation membranes were washed twice with PBS^−/−^. Subsequently, HCASMC were seeded on these membranes at a density of 2 × 10^5^/membrane. Cells were cultured for 2–3 days to obtain a confluent monolayer and starved on SMCBM (0.5% FBS) overnight prior to flow experiments. For shear stress experiments membranes were placed on top of the ATOR-coated polymer blend PLLA/P(4HB) and embedded into a parallel plate flow chamber system (FCS; Provitro GmbH, Berlin, Germany). After perfusion with cell culture medium (SMCBM + 2% FBS) for 3 h, HCASMC were harvested and prepared for RNA isolation.

### 3.8. RNA Isolation, cDNA Synthesis, and qRT-PCR

Total RNA was isolated using the RNeasy Mini Kit (Qiagen, Hilden, Germany). Reverse transcription was carried out with 1000 ng of total RNA by random hexamer primers and TaqMan reverse transcription reagents (Applied Biosystems, Carlsbad, CA, USA). The synthesized single strand cDNA was used to quantify 18S, HMG-CoA reductase, and OATP2B1 cationic transporter expression levels with a CFX96 Touch™ Real-Time PCR Detection System (Bio-RAD, Munich, Germany) using the following TaqMan gene expression assays (life technologies, Carlsbad, CA, USA): 18S (Hs03003631_g1), HMG-CoA reductase (Hs00168352_m1), and OATP2B1 (Hs01030343_m1), and MKI67 (Hs01032443_m1). Relative gene expression data were normalized to the amount of the housekeeping gene 18S in the same cDNA preparation, by using the 2^−∆∆*C*t^ method.

### 3.9. Data Collection and Statistical Analysis

All experiments were repeated at least three times and representative experiments are shown. If not indicated otherwise, means and standard deviations (SD) were analyzed using Prism 5 software (GraphPad©, La Jolla, CA, USA). A Two-Way mixed ANOVA was used to determine main effects of cell type, ATOR treatment, and polymers on alterations and interactions in viability, proliferation and ATOR-uptake. Post hoc analysis was done with adjustment for multiple testing to determine differences between groups. Statistical differences between controls and treatments normalized to a hypothetical value were calculated using an unpaired two-tailed *t*-test. Unless otherwise stated, *p* < 0.05 was defined as statistically significant. Normalized response IC_50_ curves were calculated using GraphPad© Prism 5 software (La Jolla, CA, USA).

## 4. Conclusions

As described above statins lose their supportive effects on ECs when given in high dosages [43,44,45]; keeping the alteration in IC_50_ values, and the reduced ATOR-uptake by HCASMCs in mind, one might speculate that this might limit the suitability of ATOR as drug released from biodegradable stent polymers. In our study, we observed a predominant effect of the polymer surfaces on viability and proliferation of arterial ECs, rather than a beneficial effect of the administered statin. Indeed, our previous studies [19,20] suggested, that EC function is critically affected by the stent surface polymer itself. These findings led us to the idea that cell-material interactions are able to influence the effect of drugs administered either systemically or released locally. How polymeric surfaces influence cellular gene expression and drug effects in detail, however, remains to be determined.

Apart from their lipid-lowering effects, statins have various other biological effects. These effects include the inhibition of SMC growth and the improvement of EC function. Randomized trials have consistently shown that statins lower the rate of myocardial infarction, stroke, and cardiovascular death [57]. Lately, DES have been developed with the idea of combining the principle of mechanical scaffolding with local release of effective drugs to reduce SMC proliferation subsequently attenuating restenosis [58]. However, new generation DES facilitating the control of SMC growth resulted in another problem, which should not be underestimated: stent thrombosis. Recent work links stent thrombosis to insufficient re-endothelialisation of the stented segment and to adverse platelet-material interactions. Our *in vitro* study also strengthens the hypothesis, that the stent material is able to alter cellular characteristics with the result of unintended drug effects.

However, in this *in vitro* study, we only investigated one statin and three potentially relevant biodegradable polymers based on PLLA and P(4HB). Further studies—including a wider variety of polymers and other drugs—are planned, in order to secure a more detailed understanding of how polymers influence the impact of matrix-moderated effects on cellular gene expression and drug response.

Given the potential pathophysiological relevance, the interactions of matrix- and drug-moderated effects on vascular cells should be taken more seriously into account when it comes to therapeutic applications. Our study demonstrates the influence of the polymeric surface on dose-response to drugs, drug uptake, and gene expression in HCAEC as well as in HCASMC. The actual relevance of these data for *in vivo* performance of newly developed stents is, however, the subject of future research. Taken together, these findings result in the insight that current and future stent designs should consider the differential effect of the applied polymers and loaded drugs on the targeted cells. Adjusting the chemical and mechanical requirements to appropriate physiological properties will increase the potential of biocompatible synthetic and natural polymeric materials for use both as implants and coating matrices.

## Supplementary Materials

Supplementary materials can be found at http://www.mdpi.com/1422-0067/17/2/148/s1.

## References

[1] Marks A.R. (2003). Sirolimus for the prevention of in-stent restenosis in a coronary artery. N. Engl. J. Med..

[2] Farb A., Burke A.P. (2003). Pathological mechanisms of fatal late coronary stent thrombosis in humans. Circulation.

[3] Schmidt T., Iakovou I. (2005). Incidence, predictors, and outcome of thrombosis after successful implantation of drug-eluting stents. JAMA.

[4] Joner M., Finn A.V., Farb A., Mont E.K., Kolodgie F.D., Ladich E., Kutys R., Skorija K., Gold H.K., Virmani R. (2006). Pathology of drug-eluting stents in humans: Delayed healing and late thrombotic risk. J. Am. Coll. Cardiol..

[5] Lanzer P., Sternberg K., Schmitz K.-P., Kolodgie F., Nakazawa G., Virmani R. (2008). Drug-eluting coronary stent very late thrombosis revisited. Herz.

[6] Lüscher T.F., Steffel J., Eberli F.R., Joner M., Nakazawa G., Tanner F.C., Virmani R. (2007). Drug-eluting stent and coronary thrombosis: Biological mechanisms and clinical implications. Circulation.

[7] Vorpahl M., Virmani R., Ladich E., Finn A.V. (2009). Vascular remodeling after coronary stent implantation. Minerva Cardioangiol..

[8] Jordan S.W., Chaikof E.L. (2007). Novel thromboresistant materials. J. Vasc. Surg..

[9] Sternberg K., Grabow N., Petersen S., Weitschies W., Harder C., anHarder C., Kroemer H.K., Schmitz K.-P. (2013). Advances in coronary stent technology-active drug-loaded stent surfaces for prevention of restenosis and improvement of biocompatibility. Curr. Pharm. Biotechnol..

[10] Curcio A., Torella D., Cuda G., Coppola C., Faniello M.C., Achille F., Russo V.G., Chiariello M., Indolfi C. (2004). Effect of stent coating alone on *in vitro* vascular smooth muscle cell proliferation and apoptosis. Am. J. Physiol. Heart Circ. Physiol..

[11] Nebeker J.R., Virmani R., Bennett C.L., Hoffman J.M., Samore M.H., Alvarez J., Davidson C.J., McKoy J.M., Raisch D.W., Whisenant B.K. (2006). Hypersensitivity cases associated with drug-eluting coronary stents: A review of available cases from the research on adverse drug events and reports (radar) project. J. Am. Coll. Cardiol..

[12] Curcio A., Torella D., Indolfi C. (2011). Mechanisms of smooth muscle cell proliferation and endothelial regeneration after vascular injury and stenting: Approach to therapy. Circ. J..

[13] Strohbach A., Busch R. (2015). Polymers for cardiovascular stent coatings. Int. J. Polym. Sci..

[14] Ge J., Qian J., Wang X., Wang Q., Yan W., Yan Y., Fan B., Ge L., Liu X. (2007). Effectiveness and safety of the sirolimus-eluting stents coated with bioabsorbable polymer coating in human coronary arteries. Catheter. Cardiovasc. Interv..

[15] Pan C.J., Tang J.J., Weng Y.J., Wang J., Huang N. (2007). Preparation and characterization of rapamycin-loaded plga coating stent. J. Mater. Sci. Mater. Med..

[16] Von Haehling S. (2009). Statins for heart failure: Still caught in no man’s land?. Clin. Sci..

[17] Bünger C.M., Grabow N., Sternberg K., Goosmann M., Schmitz K.P., Kreutzer H.J., Ince H., Kische S., Nienaber C.A., Martin D.P. (2007). A biodegradable stent based on poly(l-lactide) and poly(4-hydroxybutyrate) for peripheral vascular application: Preliminary experience in the pig. J. Endovasc. Ther..

[18] Grabow N., Martin D.P., Schmitz K., Sternberg K.-P. (2010). Absorbable polymer stent technologies for vascular regeneration. J. Chem. Technol. Biotechnol..

[19] Busch R., Strohbach A., Peterson S., Sternberg K., Felix S. (2013). Parameters of endothelial function are dependent on polymeric surface material. Biomed. Tech..

[20] Busch R., Strohbach A., Rethfeldt S., Walz S., Busch M., Petersen S., Felix S., Sternberg K. (2014). New stent surface materials: The impact of polymer-dependent interactions of human endothelial cells, smooth muscle cells, and platelets. Acta Biomater..

[21] Endo A. (1992). The discovery and development of HMG-CoA reductase inhibitors. J. Lipid Res..

[22] LaRosa J.C., He J., Vupputuri S. (1999). Effect of statins on risk of coronary disease: A meta-analysis of randomized controlled trials. JAMA.

[23] Lahera V., Goicoechea M., de Vinuesa S.G., Miana M., de las Heras N., Cachofeiro V., Luno J. (2007). Endothelial dysfunction, oxidative stress and inflammation in atherosclerosis: Beneficial effects of statins. Curr. Med. Chem..

[24] Jakóbisiak M., Bruno S., Skierski J.S., Darzynkiewicz Z. (1991). Cell cycle-specific effects of lovastatin. Proc. Natl. Acad. Sci. USA.

[25] Vaughan C.J., Gotto A.M., Basson C.T. (2000). The evolving role of statins in the management of atherosclerosis. J. Am. Coll. Cardiol..

[26] Wong W.W., Tan M.M., Xia Z., Dimitroulakos J., Minden M.D., Penn L.Z. (2001). Cerivastatin triggers tumor-specific apoptosis with higher efficacy than lovastatin. Clin. Cancer Res..

[27] Laufs U. (2003). Beyond lipid-lowering: Effects of statins on endothelial nitric oxide. Eur. J. Clin. Pharmacol..

[28] Von Haehling S., Anker S.D., Bassenge E. (2003). Statins and the role of nitric oxide in chronic heart failure. Heart Fail. Rev..

[29] Liao J.K., Laufs U. (2005). Pleiotropic effects of statins. Annu. Rev. Pharmacol. Toxicol..

[30] Ito M.K., Talbert R.L., Tsimikas S. (2006). Statin-associated pleiotropy: Possible beneficial effects beyond cholesterol reduction. Pharmacotherapy.

[31] Eindhoven J.A., Onuma Y., Oemrawsingh R.M., Daemen J., van Nierop J.W.I., de Jaegere P.P.T., Boersma E., Serruys P.W., van Domburg R.T. (2012). Long-term outcome after statin treatment in routine clinical practice: Results from a prospective PCI cohort study. EuroIntervention.

[32] Matsushima S., Maeda K., Kondo C., Hirano M., Sasaki M., Suzuki H., Sugiyama Y. (2005). Identification of the hepatic efflux transporters of organic anions using double-transfected madin-darby canine kidney ii cells expressing human organic anion-transporting polypeptide 1B1 (OATP1B1)/multidrug resistance-associated protein 2, OATP1B1/multidrug resistance 1, and OATP1B1/breast cancer resistance protein. J. Pharmacol. Exp. Ther..

[33] Grube M., Köck K., Oswald S., Draber K., Meissner K., Eckel L., Böhm S.B., Felix M., Vogelgesang S., Jedlitschky G. (2006). Organic anion transporting polypeptide 2B1 is a high-affinity transporter for atorvastatin and is expressed in the human heart. Clin. Pharmacol. Ther..

[34] Guijarro C., Blanco-Colio L.M., Ortego M., Alonso C., Ortiz A., Plaza J.J., Díaz C., Hernández G., Egido J. (1998). 3-Hydroxy-3-methylglutaryl coenzyme a reductase and isoprenylation inhibitors induce apoptosis of vascular smooth muscle cells in culture. Circ. Res..

[35] Corsini A., Pazzucconi F., Arnaboldi L., Pfister P., Fumagalli R., Paoletti R., Sirtori C.R. (1998). Direct effects of statins on the vascular wall. J. Cardiovasc. Pharmacol..

[36] Briguori C., Colombo A., Airoldi F., Violante A., Focaccio A., Balestrieri P., Elia P.P., Golia B., Lepore S., Riviezzo G. (2004). Statin administration before percutaneous coronary intervention: Impact on periprocedural myocardial infarction. Eur. Heart J..

[37] Petersen S., Hussner J., Reske T., Grabow N., Senz V., Begunk R., Arbeiter D., Kroemer H.K., Schmitz K.-P., Meyer Zu Schwabedissen H.E. (2013). *In vitro* study of dual drug-eluting stents with locally focused sirolimus and atorvastatin release. J. Mater. Sci. Mater. Med..

[38] Lee C.-H., Chang S.-H., Lin Y.-H., Liu S.-J., Wang C.-J., Hsu M.-Y., Hung K.-C., Yeh Y.-H., Chen W.-J., Hsieh I.-C. (2014). Acceleration of re-endothelialization and inhibition of neointimal formation using hybrid biodegradable nanofibrous rosuvastatin-loaded stents. Biomaterials.

[39] Zago A.C., Matte B.S., Reginato L., Iturry-Yamamoto G., Krepsky A., Carlos L., Bergoli C., Balvedi J., Raudales J.C., Saadi E.K. (2012). First-in-man study of simvastatin-eluting stent in de novo coronary lesions. Circ. J..

[40] Rüder C., Sauter T., Kratz K., Peter J., Jung F., Lendlein A., Zohlnhöfer D. (2012). Smooth muscle and endothelial cell behaviour on degradable copolyetheresterurethane films. Clin. Hemorheol. Microcirc..

[41] Turner N.A., Midgley L., O’Regan D.J., Porter K.E. (2007). Comparison of the efficacies of five different statins on inhibition of human saphenous vein smooth muscle cell proliferation and invasion. J. Cardiovasc. Pharmacol..

[42] Kruger P.S., Freir N.M., Venkatesh B., Robertson T.A., Roberts M.S., Jones M. (2009). A preliminary study of atorvastatin plasma concentrations in critically ill patients with sepsis. Intensive Care Med..

[43] Frick M., Dulak J., Cisowski J., Józkowicz A., Zwick R., Alber H., Dichtl W., Schwarzacher S.P., Pachinger O., Weidinger F. (2003). Statins differentially regulate vascular endothelial growth factor synthesis in endothelial and vascular smooth muscle cells. Atherosclerosis.

[44] Weis M., Heeschen C., Glassford A.J., Cooke J.P. (2002). Statins have biphasic effects on angiogenesis. Circulation.

[45] Urbich C., Dernbach E., Zeiher A.M., Dimmeler S. (2002). Double-edged role of statins in angiogenesis signaling. Circ. Res..

[46] Wells R.G. (2008). The role of matrix stiffness in regulating cell behavior. Hepatology.

[47] Duprez D.A. (2010). Arterial stiffness and endothelial function: Key players in vascular health. Hypertension.

[48] Kolahi K.S., Donjacour A., Liu X., Lin W., Simbulan R.K., Bloise E., Maltepe E., Rinaudo P. (2012). Effect of substrate stiffness on early mouse embryo development. PLoS ONE.

[49] Chen G., Zhao L., Feng J., You G., Sun Q., Li P., Han D., Zhou H. (2013). Validation of reliable reference genes for real-time PCR in human umbilical vein endothelial cells on substrates with different stiffness. PLoS ONE.

[50] Levental I., Georges P.C., Janmey P.A. (2007). Soft biological materials and their impact on cell function. Soft Matter.

[51] Rehfeldt F., Engler A.J., Eckhardt A., Ahmed F., Discher D.E. (2007). Cell responses to the mechanochemical microenvironment–implications for regenerative medicine and drug delivery. Adv. Drug Deliv. Rev..

[52] Engler A.J., Sen S., Sweeney H.L., Discher D.E. (2006). Matrix elasticity directs stem cell lineage specification. Cell.

[53] Grabow N., Bünger C.M., Schultze C., Schmohl K., Martin D.P., Williams S.F., Sternberg K., Schmitz K.-P. (2007). A biodegradable slotted tube stent based on poly(l-lactide) and poly(4-hydroxybutyrate) for rapid balloon-expansion. Ann. Biomed. Eng..

[54] Griffin M.A., Sen S., Sweeney H.L., Discher D.E. (2004). Adhesion-contractile balance in myocyte differentiation. J. Cell Sci..

[55] Ross W., Hall P.A. (1995). Ki67: From antibody to molecule to understanding?. Clin. Mol. Pathol..

[56] Sharma S., Christopoulos C., Kukreja N., Gorog D.A. (2011). Local drug delivery for percutaneous coronary intervention. Pharmacol. Ther..

[57] Ludman A., Venugopal V., Yellon D.M., Hausenloy D.J. (2009). Statins and cardioprotection—More than just lipid lowering?. Pharmacol. Ther..

[58] Prasad K. (2013). Do statins have a role in reduction/prevention of post-PCI restenosis?. Cardiovasc. Ther..

